# The involvement of FoxO in cell survival and chemosensitivity mediated by Mirk/Dyrk1B in ovarian cancer

**DOI:** 10.3892/ijo.2011.1293

**Published:** 2011-12-12

**Authors:** JINGCHUN GAO, XIANGJUN YANG, PING YIN, WENFENG HU, HONGFENG LIAO, ZHIHUI MIAO, CHAO PAN, NA LI

**Affiliations:** 1 Department of Obstetrics and Gynecology, First Affiliated Hospital, Dalian Medical University, Dalian, Liaoning 116011; 2 Department of Obstetrics and Gynecology, Zhongshan Hospital Xiamen University, Xiamen, Fujian 361004, P.R. China; 3 Department of Pathology, Zhongshan Hospital Xiamen University, Xiamen, Fujian 361004, P.R. China

**Keywords:** minibrain-related kinase/dual specificity tyrosine-phosphorylation-regulated kinase 1B, forkhead subclass O, apoptosis, ovarian cancer

## Abstract

Minibrain-related kinase (Mirk) is a serine/threonine kinase which is also known as the dual specificity tyrosine-phosphorylation-regulated kinase 1B (Dyrk1B). It is known that Dyrk1A, the closest family member to Mirk/Dyrk1B can mediate cellular localization of mammalian forkhead subclass O (FoxO1), a transcription factor, although the effect of Mirk/Dyrk1B on FoxO factors remains to be defined. In this study, we showed that Mirk/Dyrk1B protein was overexpressed in 5 of 8 ovarian cancer cell lines and negatively correlated with the protein level of FoxO factors (FoxO1+FoxO3A). Knockdown of Mirk by small interfering RNA (siRNA) resulted in cell apoptosis and sensitized cells to cisplatin accompanied by nuclear translocation of FoxO1 and/or FoxO3A as well as increased Bim, TRADD, cleaved caspase-3 and PARP. Furthermore, combined siRNAs of Mirk with FoxO1 and/or FoxO3A led to fewer apoptotic cells and cisplatin sensitivity compared to Mirk siRNA alone, suggesting that FoxO is involved in Mirk-mediated cell survival and chemosensitivity of ovarian cancer. Taken together, Mirk/Dyrk1B plays an important role in ovarian cancer cell survival through modulating FoxO translocation and may be a novel therapeutic target for ovarian cancer.

## Introduction

Ovarian cancer is the fourth most common cause of cancer deaths in women exceeded only by breast, colon and lung malignancies with the majority of cases being diagnosed after the disease has become metastatic, and the 5-year survival is about 40% (1). Although the chemotherapeutic agents, such as cisplatin, carboplatin and paclitaxel have been known to be effective against ovarian carcinomas, the efficacy of which is limited by intrinsic or acquired chemoresistance in residual cells. Therefore, it is warranted to explore new therapeutic target for further treatment and reducing recurrence of the disease.

Minibrain-related kinase (Mirk) is a serine/threonine kinase which is also known as the dual specificity tyrosine-phosphorylation-regulated kinase 1B (Dyrk1B). Mirk/Dyrk1B is one of members of Dyrk family which have the ability to auto-phosphorylate on tyrosine and then phosphorylate other substrates on serine and threonine (2); therefore, they are categorized as dual function kinases. Mirk/Dyrk1B is expressed in few normal tissues, but in many types of human cancer (3), such as sarcomas (4,5), pancreatic and colon carcinomas (6), and cervical cancer (7). Our recent study also found Mirk/Dyrk1B was overexpressed in a wide spectrum of cell lines and tumor specimens of lung cancer (8). Furthermore, the knockdown Mirk/Dyrk1B by small interfering RNA (siRNA) induced cell apoptosis and increased sensitivity of human cancer cells to conventional chemotherapeutics *in vitro* (5,6,8). Our previous results also showed Mirk/Dyrk1B function in an orthotopic mouse model (8). Moreover, a study in osteosarcoma demonstrates that the overall survival rate of patients is negatively correlated with the levels of Mirk/Dyrk1B protein expression (5). All of the above suggest that Mirk/Dyrk1B could serve as a novel therapeutic target and the overexpressed Mirk/Dyrk1B may be a diagnostic marker and survival factor for various types of human cancer.

The mammalian forkhead subclass O (FoxO) family members of transcriptional factors, such as FoxO1 (FHKR) and FoxO3a (FKHR-L1) are characterized by a distinctive forkhead DNA binding domain which function downstream of PI3K antagonist PTEN in cancer cells, inhibit cell cycle progression and promote cell death by modulating the expression of genes encoding apoptosis (9), growth regulatory proteins (10) and stress response (11,12). The modulating mechanisms include: a) direct binding to the insulin response sequence (IRS) in gene promoters (e.g., apoptotic proteins Bim and fas ligand) and b) tethering to the other transcription factors (cell cycle regulators cyclin G2 and cyclin D1). The phosphorylation of FoxO factors by protein kinases, such as Akt, serum and glucocorticoid inducible kinase (SGK) leads to their translocation from the nucleus to the cytoplasm and loss of proapoptotic function due to inactivation (13,14). Whereas, the unphosphorylated active forms of FoxO reside in the nucleus and induces cell death by up-regulation of apoptotic proteins, such as Bim, p27, TRADD (15–17) and repression of antiapoptotic molecule FLIP and Bcl-XL (18,19). Furthermore, Dyrk1A, the closest family member to Mirk/Dyrk1B, has been found to phosphorylate FoxO1 at ser329, a novel *in vivo* phosphorylation site (20), and mediates cellular localization of FoxO1 in immortalized cells (21). More recently, the serine/threonine kinase Mirk/Dyrk1B has been thought to be a transcriptional co-activator which increases expression of a cohort of antioxidants in human cancer cells (22,23). In addition, both FoxO1 and FoxO3a have been reported to be involved in cytotoxic stress and drug-resistance induced by chemotherapeutics in ovarian cancers (24,25). Taken together, we hypothesize that FoxO factors may be a novel downstream manner by which Mirk/Dyrk1B serves as an antiapoptotic factor and contribute to ovarian cancer cell survival.

Although a few studies show that Mirk/Dyrk1B mediates ovarian cancer cell survival, in particular for quiescent tumor cells, and depleting Mirk kinase increase cisplatin toxicity associated with higher level of reactive oxygen species (ROS) in ovarian cancer cells (23,26), insufficient data regarding the effect of Mirk/Dyrk1B on human ovarian cancer cells are available, and the mechanisms involved remain unclear. In this study, we have identified that Mirk/Dyrk1B is overexpressed in a wide spectrum of ovarian cancer cell lines and primary tumors in which it is located in the cytoplasm. Mirk/Dyrk1B-mediated cell survival and chemosensitivity is correlated with expression and nuclear translocation of FoxO1 and/or FoxO3A in ovarian cancer.

## Materials and methods

### Antibodies

The rabbit polyclonal Dyrk1B antibody (C-term, AP7538b) was purchased from Abgent (San Diego, CA). Anti-Bim, anti-TRADD, and goat anti-mouse IgG horseradish peroxidase (HRP)-conjugated secondary antibody were purchased from Santa Cruz Biotechnology (Santa Cruz, CA). Anti-FKHR/FoxO1, anti-caspase-3, and anti-poly(ADP-ribose) polymerase (PARP) were purchased from Cell Signaling Technology (Danvers, MA). Anti-FKHR-L1/FoxO3a was purchased from Upstate (Lake Placid, NY). Alexa Fluor 594 F(ab′) fragment of goat anti-mouse IgG were purchased from Invitrogen (Eugene, OR). Anti-β-actin and donkey anti-rabbit IgG HRP-conjugated secondary antibody were purchased from Sigma (St. Louis, MO) and Amersham Biosciences (Piscataway, NJ), respectively.

### Cell lines and cell culture

Human ovarian cancer cell lines used were OV2008, OVCAR3, OVCAR5, SKOV3, MDAH2774, OVCAR10, OV1063, OVCAR8. The SKOV3 and OVCAR3 were purchased from American Type Culture Collection (Manassas, VA); others were gifts from Dr Jin Q. Cheng (H. Lee Moffitt Cancer Center and Research Institute, USA). All lines were maintained in DMEM supplemented with 10% heat-inactivated (56˚C, 30 min) fetal bovine serum (FBS; Invitrogen, Grand Island, NY). Monolayer cultures were incubated at 37˚C in a 95% humidified atmosphere air containing 5% CO_2_.

### Small interfering RNA treatment

Cells were reverse-transfected with small interfering RNAs (siRNAs) using Lipofectamine 2000 transfection reagent (Invitrogen) according to the manufacturer's instructions. The Mirk/Dyrk1B, FoxO1 and FoxO3a siRNA duplexes as well as the corresponding non-specific control siRNA duplexes as described (8,27) were supplied by Dharmacon and Ambion, respectively. After a 72-h incubation or at indicated time points, cells were harvested or trypsinized and replated for subsequent experiments.

### Flow cytometry analysis

After 72-h treatment with siRNAs, cells were subjected to flow cytometry analyses of apoptosis. Apoptosis was assayed using Pharmingen (San Diego, CA) PE-conjugated monoclonal active caspase-3 antibody apoptosis kit without modification as described previously (8). A total of 10,000 cells per experimental condition were used for processing and analysis of fluorescence on Becton-Dickinson FACScan (BD, Franklin Lakes, NJ) using CellQuest software. Apoptosis of siRNA-transfected cells after 48-h exposure to the chemotherapeutic agent cisplatin (CDDP) was also detected by flow cytometry analysis.

### Western blot analysis

Cells were washed twice with cold PBS and lysed with buffer A [10 mM Tris-HCl (pH 7.4), 1% Triton X-100, 0.1% SDS, 150 mM NaCl, 1 mM EDTA, 1 mM dithiothreitol, 0.5 mM phenylmethylsulfonyl fluoride, 10 μg/ml leupeptin, 5 μg/ml aprotinin]. After incubation for 30 min on ice, the suspensions were centrifuged (15,000 g for 30 min). The supernatants were removed and stored at −80˚C until analysis using gel electrophoresis. The protein concentration was determined by Bio-Rad protein estimation assay according to the manufacturer's instructions. For Western blot analysis, ~60–100 μg of whole cell proteins were separated using 10% or 12% SDS-PAGE and transferred to nitrocellulose membranes. After blocking of the membranes with 10 mM Tris-HCl (pH 7.4), 150 mM NaCl, and 0.1% Tween-20 containing 5% non-fat dry milk at room temperature for 60 min, the membranes were incubated with indicated antibodies at 4˚C overnight and then with the HRP-conjugated secondary anti-rabbit or anti-mouse antibodies at room temperature for 60 min. Each protein was detected using the enhanced chemiluminescence (Amersham Biosciences) system. β-actin was used as an internal control.

### Patients and tumor specimens

The primary human ovarian cancer specimens were obtained from 51 patients who underwent surgery without chemotherapy or radiation prior to resection at the First Affiliated Hospital of Dalian Medical University and Zhongshan Hospital Xiamen University between 1996 and 2010. Each sample contained at least 80% tumor cells, confirmed by microscopic examination. As control groups, the specimens obtained from 16 patients with ovarian benign tumor and 9 cases of non-neoplastic cyst were also examined. The clinicopathological aspects of all samples were listed in Table I. The study was approved by the Research Committee of the First Affiliated Hospital of Dalian Medical University and Zhongshan Hospital Xiamen University.

### Immunostaining analysis of Mirk/Dyrk1B and FoxO

Immunohistochemistry staining using anti-Dyrk1B as the primary antibody. After antigen retrieval with citrate, the endogenous peroxidase activity was blocked by incubation with 0.3% hydrogen peroxide. Slides were incubated overnight with 1:50 primary antibody at 4˚C. Antigen-antibody complexes were detected by the avidin-biotin peroxidase method using ABC Kit (Vector Laboratories, Inc., Burlingame, CA) and DAB (Dako, Japan) reagents. Sections were counterstained with hematoxylin and viewed using a microscope (Zeiss, Tokyo, Japan). For immunofluorescent staining, cells were fixed with 4% paraformaldehyde for 20 min on ice. Cells were incubated in 1% bovine serum albumin (BSA) in PBS for 30 min. Primary antibody against FoxO1 or FoxO3a (1:100) was added in 1% BSA/PBS for overnight at 4˚C. After washing, cells were incubated with Alexa Fluor 594 F(ab′) fragment of goat anti-mouse IgG for 30 min at room temperature, and nuclei were then counterstained with DAPI allowing visualization of nuclei with a Leica Confocal Microscope System.

### Statistical analysis

Each experiment was repeated three times. Data are presented as mean ± SD. StatView 5.0 software was used for statistical analyses. Statistical comparison between control and experimental groups were performed using χ^2^ test (for incidence only) and Student's t-test. The correlations between Mirk expression and FoxO were analyzed by simple regression. Differences were considered to be statistically significant when P<0.05.

## Results

### Mirk is widely overexpressed in ovarian cancer cells and correlates with FoxO expression

**Figure 1:**
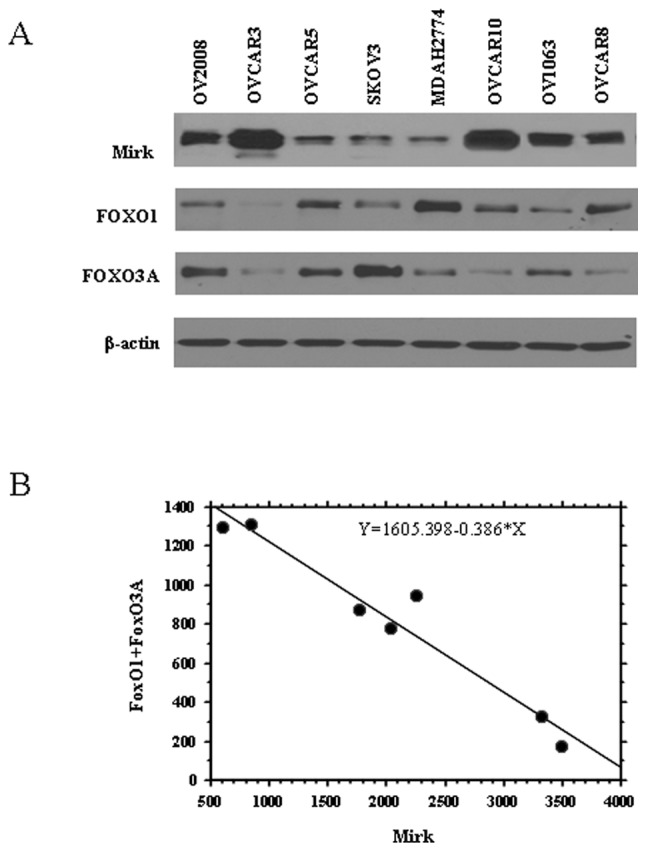
Mirk is overexpressed in a wide variety of ovarian cancer cell lines and correlated with FoxO expression. (A), Western blot analysis of Mirk (69 and 71 kDa), FoxO1 (70 kDa) and FoxO3A (97 kDa) in ovarian cancer cells. Equal loading and transfer were shown by repeat probing with β-actin (42 kDa). (B), The correlation between the expression of Mirk protein and the total level of FoxO (FoxO1+FoxO3A) was analyzed by simple regression.

In this study, we first evaluated protein expression of Mirk in 8 human ovarian cancer cell lines. The 8 cell lines all expressed Mirk protein, 5 of them with high levels (Fig. 1A), which is consistent with the findings reported by Hu *et al* (26). Based on the hypothesis described above that the FoxO transcriptional factors may be involved in Mirk function in ovarian cancer, we further examined the expression of both FoxO1 and FoxO3A in the 8 cell lines (Fig. 1A). As shown in Fig. 1B, correlation appears to be negative between the expression of Mirk protein and the total level of FoxO (FoxO1+FoxO3A) in ovarian cancer cells (R^2^=0.946 and P<0.001), suggesting FoxO1 and/or FoxO3A may be associated with Mirk function or kinase activity.

### Knockdown of Mirk induces apoptosis involving the downstream signals of FoxO and results in chemosensitivity in vitro

**Figure 2:**
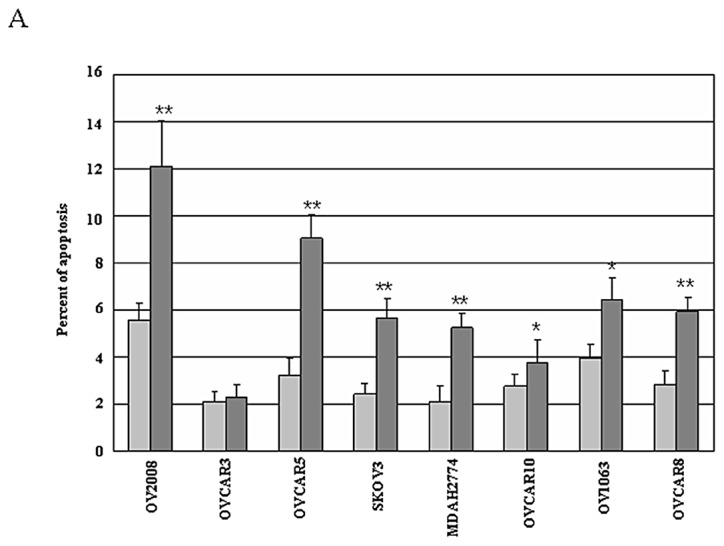

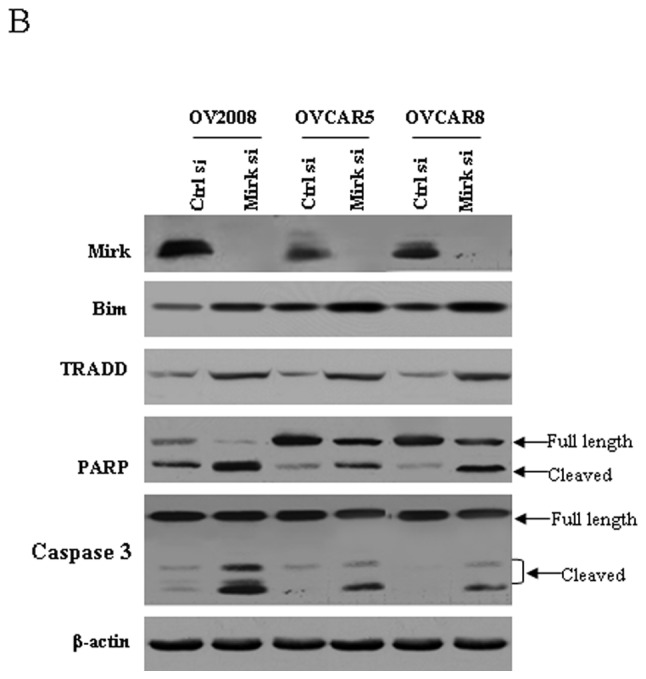

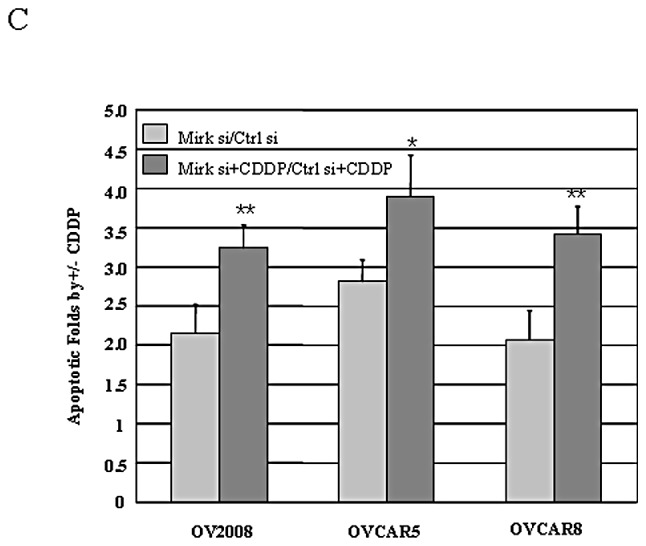
Knockdown of Mirk induces apoptosis involving the downstream signals of FoxO and results in chemosensitivity in ovarian cancer cells. The cells were exposed to siRNAs (20 nM) for 72 h, (A) percentage of cell apoptosis evidenced by positive cells of active caspase-3 in the cells was assessed by flow cytometry analysis, (B) followed by measurement of Mirk (69 and 71 kDa), Bim (28 kDa), TRADD (34 kDa), PARP (full length 116 kDa and cleaved 89 kDa), and caspase-3 (full length 35 kDa and cleaved 17 kDa) proteins by Western blot analysis with equal loading and transfer shown by repeat probing with β-actin (42 kDa), and (C) cells were collected and plated in 6-well plates and allowed to grow for ~18–24 h before 48-h exposure to 10 μM cisplatin, then cell apoptosis (fold of samples by siRNA alone) evidenced by positive cells of active caspase-3 in the cells was assessed by flow cytometry analysis. ^*^P<0.05; ^**^P<0.001 compared with control.

We have reported the concentration- and target-dependent effects on Mirk protein and apoptosis occurred in lung cancer cells induced by Mirk siRNA (~5–20 nM) and the corresponding individual siRNAs #1-#4 of Mirk (8). In this study, we examined the consequence of Mirk knockdown using 20 nM siRNA duplexes #4 targeting Mirk. We exposed 8 ovarian cancer cell lines to Mirk siRNA for 72 h followed by assessment of cellular apoptosis. As shown in Fig. 2A, Mirk knockdown by siRNA resulted in cellular apoptosis (~1.05- to 2.81-fold of control), as evidenced by more Mirk siRNA-treated cells staining with cleaved caspase-3. Intriguingly, the Mirk knockdown-induced apoptosis in variant cell lines appears to be positively correlated with FoxO (FoxO1+FoxO3A) expression. To investigate the mechanisms involved in apoptosis induced by Mirk siRNA, the downstream signals of FoxO factors were determined in a representative panel of three ovarian cancer cell lines by Western blot analysis. As shown in Fig. 2B, exposure of these cell lines to Mirk siRNA for 72 h was associated with knockdown of Mirk, cleavage of caspase-3 and PARP, compared with that shown with control siRNA, and resulted in upregulation of pro-apoptotic Bim and TRADD in all three cell lines, suggesting knockdown Mirk-induced cellular apoptosis may be associated with FoxO factors as well as their downstream signals. We next investigated the effects of constitutively expressed Mirk on sensitivity of ovarian cancer cells to conventional chemotherapeutics, Mirk siRNA-treated OV2008, OVCAR5, and OVCAR8 cells were exposed to indicated dose of cisplatin for apoptosis assays. Mirk siRNA treatment and exposure to cisplatin in these cells resulted in increased apoptosis (measured in fold) compared with cells treated with control siRNA by caspase-3 assay (Fig. 2C), indicating that knockdown of Mirk sensitizes ovarian cancer cells to chemotherapy-induced apoptosis.

### Mirk modulates cell survival associated with nuclear translocation of FoxO

**Figure 3:**
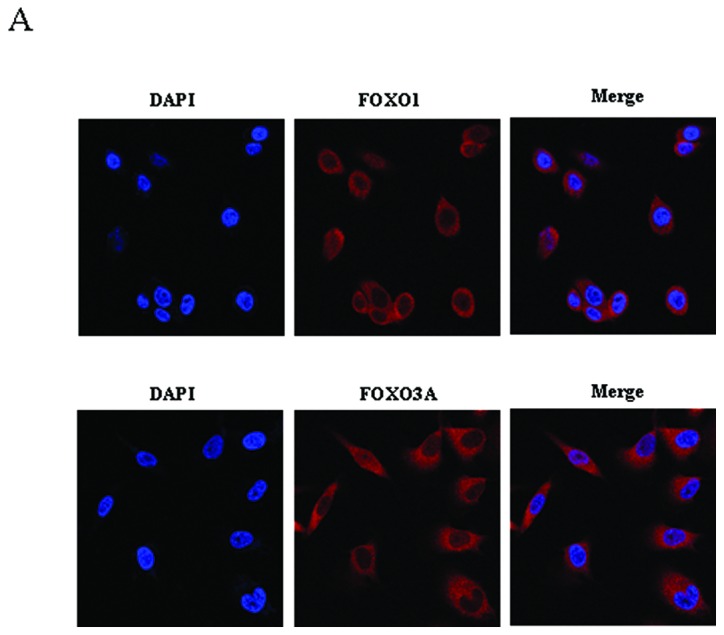

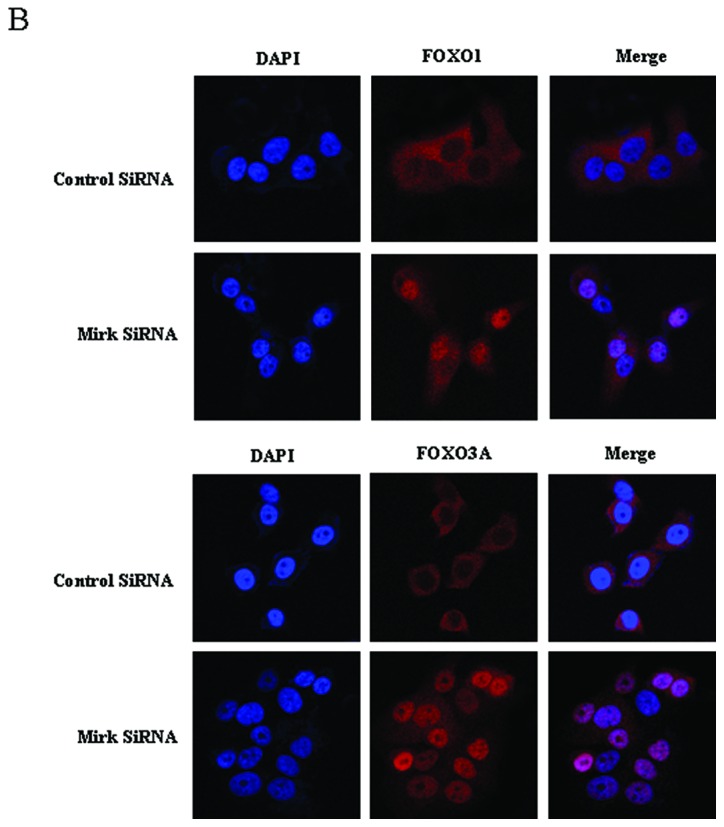

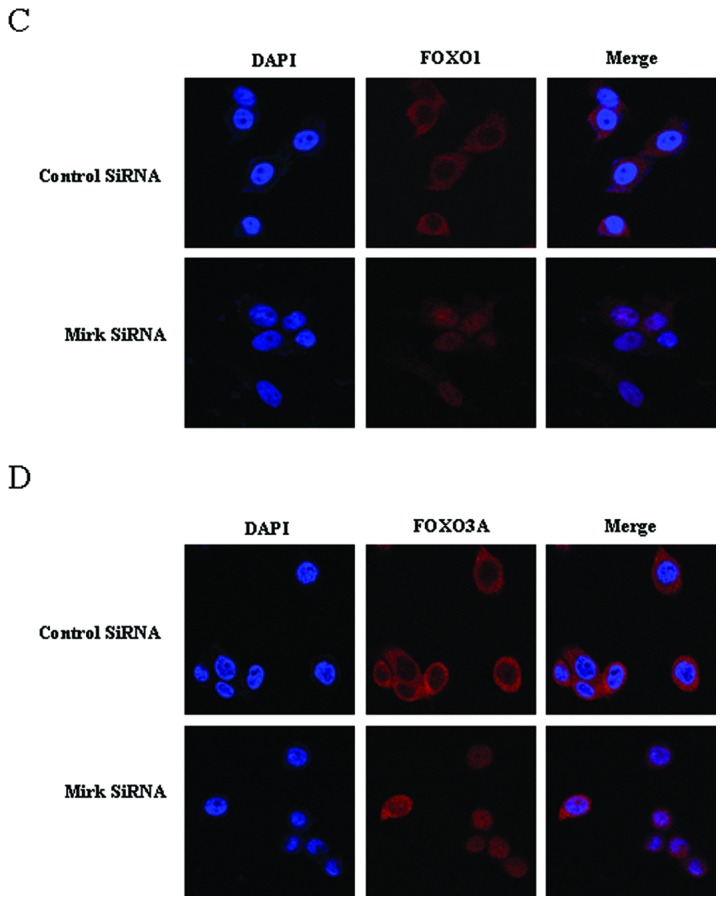
Confocal microscopy of FoxO1 or FoxO3A expression in Mirk-modulated cell survival in ovarian cancer cells. (A), Both panels show the nuclei labeled with 4′,6-diamidine-2-phenylindole (DAPI, blue), FoxO1 or Foxo3a was cytoplasmic visualized with the use of Alexa 594 (red), and the merge of DAPI and FoxO1 or FoxO3A, respectively. (B), Upper panels show DAPI, FoxO1, and the merge of DAPI and FoxO1 with/without Mirk knockdown in OVCAR5 cells. Bottom panels show DAPI, FoxO3A, and the merge of DAPI and FoxO3A with/without Mirk knockdown in OVCAR5 cells. (C), Both panels show DAPI, FoxO1, and the merge of DAPI and FoxO1 with/without Mirk knockdown in OVCAR8 cells. (D), Both panels show DAPI, FoxO1, and the merge of DAPI and FoxO3A with/without Mirk knockdown in OV2008 cells.

As described above, the phosphorylation of FoxO factors leads to their translocation from the nucleus to the cytoplasm and loss proapoptotic function due to inactivation. Whereas, the unphosphorylated active forms of FoxO reside in the nucleus and induces cellular apoptosis. Previous studies also demonstrate Dyrk1A, the closest isoform of Mirk may phosphorylate FoxO1 and promote nuclear output (20,21), thus there is a possibility that it is the subcellular localization and phosphorylation of FoxO factors but not the total protein level that is altered in Mirk/Dyrk1B siRNA-treated ovarian cancer cells. To explore this hypothesis, both FoxO1 and FoxO3A were detected by immunofluorescent staining in the cell lines OV2008, OVCAR5 or OVCAR8 treated with/without Mirk siRNA. Interestingly, both FoxO1 and FoxO3A are expressed in cytoplasm of these lines (Fig. 3A). Knockdown Mirk induced nuclear translocation of both FoxO1 and FoxO3A in OVCAR5 (Fig. 3B), of FoxO1 alone (not FoxO3A) in OVCAR8 (Fig. 3C), and of FoxO3A alone (not FoxO1) in OV2008 (Fig. 3D). Taken together, these results suggest that FoxO1 and/or FoxO3A may be a novel downstream way in which Mirk serves as an antiapoptotic factor in ovarian cancer cells.

### Knockdown FoxO results in less Mirk siRNA-induced apoptosis and decreased sensitivity to chemotherapy in ovarian cancer cells

**Figure 4:**
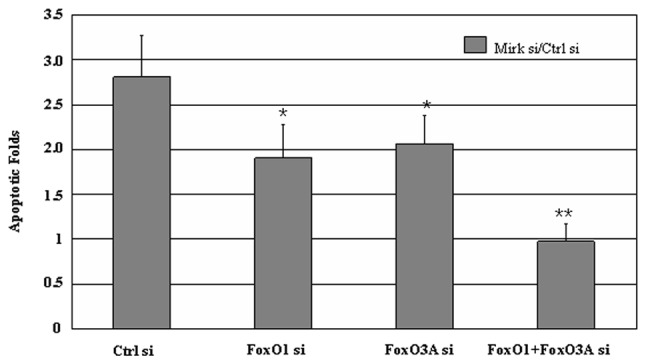
Knockdown FoxO results in less Mirk siRNA-induced apoptosis. The OVCAR5 cells were exposed to siRNAs of FoxO1 and/or FoxO3A combining with/without Mirk siRNA for 72 h, percentage of cell apoptosis evidenced by positive cells of active caspase-3 in the cells was assessed by flow cytometry analysis. ^*^P<0.05; ^**^P<0.001 compared with control.

To further determine the effects of FoxO factors on Mirk-modulated ovarian cancer cell survival, FoxO siRNAs were exposed to the cells of OV2008, OVCAR5 or OVCAR8 treated with/without Mirk siRNA for 72 h, then the apoptotic cells evidenced by cleaved caspase-3 were detected by flow cytometry analysis. Combined siRNAs of Mirk with FoxO1 and/or FoxO3A led to less cellular apoptosis than Mirk siRNA alone in all three lines (Fig. 4), and less sensitivity to chemotherapeutics (data not shown). These results suggest FoxO1 and/or FoxO3A are involved in Mirk-mediated cell survival in ovarian cancer cells.

### Mirk is overexpressed in tumor specimens from clinical ovarian cancer cases

**Figure 5:**
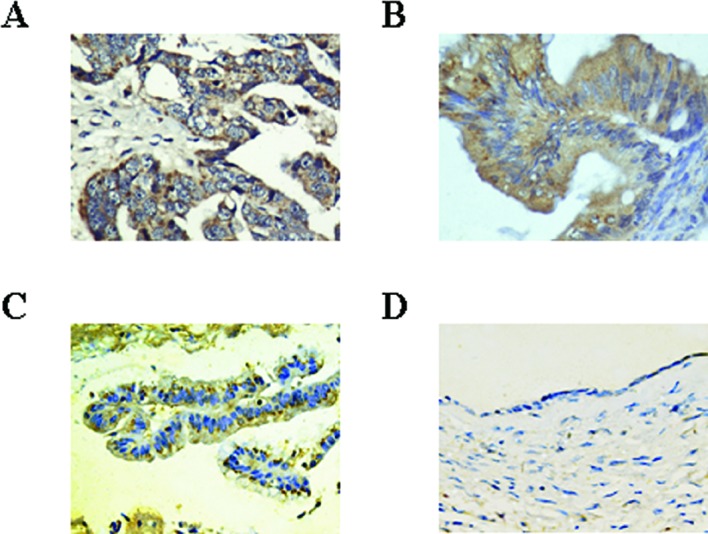
Mirk expression in ovarian cancer specimens and non-malignant ovarian tumors or cysts. The panels show immunohistological Mirk expression in formaldehyde-fixed and paraffin-embedded histologic sections of serous cystadenocarcinoma (A), mucinous cystadenocarcinoma (B), cystadenomas (C), and non-neoplastic ovarian cysts (D). The Mirk is cytoplasmic and the nuclei were counterstained with hematoxylin (original magnification ×400).

Mirk is expressed at low level in most adult tissues. We examined expression patterns of Mirk in ovarian cancer. As listed in Table I, in this study we not only examined Mirk expression in ovarian cancer specimens included 38 serous and 13 mucinous but also 16 benign cystadenomas and 9 non-neoplastic ovarian cysts by immunohistochemisty. Mirk was detected in 74.5% of ovarian cancers and overexpressed in 41% of the specimens (data not shown), and the incidence was higher than that found in both cystadenomas and non-neoplastic cysts (P<0.001 and P<0.05, respectively). We further found the overexpressed Mirk was located in the cytoplasm of ovarian cancer specimens (Fig. 5A and B) similarly to the findings in other organ cancers (4,22). Compared with Mirk expression in most of ovarian cancer specimens, it was weakly expressed only in about 37% ovarian cystadenomas (Fig. 5C) and not expressed in the ovarian non-neoplastic cysts (Fig. 5D), indicating Mirk may be associated with ovarian tumorigenesis.

## Discussion

Our study demonstrates that Mirk/Dyrk1B is overexpressed in a variety of ovarian cancer cells and clinical specimens. Knockdown of Mirk/Dyrk1B induced apoptosis of ovarian cancer cells *in vitro* and sensitized ovarian cancer cells to chemotherapeutics. These results are consistent with previous studies (3–6,8) on other types of human cancers, indicating that Mirk may be a novel therapeutic target for ovarian cancer treatment. Furthermore, we found Mirk to be expressed at higher levels in both serous and mucinous ovarian cancers than that in cystadenomas and ovarian non-neoplastic cysts. Therefore, our study along with others (23,28) suggests that Mirk may also play a role in ovarian tumorigenesis.

To date, the downstream signals of Mirk remain unclear. Given the main function of Mirk in ovarian cancer cells in mediating cell survival observed in this study, the mechanisms involved may include FoxO family members, such as FoxO1 and/or FoxO3A as well as their downstream signals, which are constitutively expressed in ovarian cancer cells (Fig. 1A). To the best of our knowledge, our study is the first to show an effect of FoxO involved in Mirk-mediated cancer cell survival. It has been reported that Dyrk1A, the closest family member to Dyrk1B/Mirk, may phosphorylate FoxO1 at ser329, a novel *in vivo* phosphorylation site (25), and promote the phosphorylated FoxO1 nuclear output (20,21). The knockdown of endogenous Dyrk1A by siRNA mediates cellular localization of FoxO1 in immortalized cells (24). Thus, we hypothesize that FoxO factors may contain a Mirk phosphorylation site, on which further study is needed. In our study, we also found that FoxO1 and/or FoxO3A nuclear translocation is concomitant with cell apoptosis induced by Mirk knockdown, which together with previous studies (24,25) suggest that it might be the subcellular localization and phosphorylation of FoxO that is altered in Mirk siRNA-treated ovarian cancer cells.

Taken together, Mirk/Dyrk1B is overexpressed in a wide spectrum of ovarian cancer cell lines and human specimens. The Mirk/Dyrk1B-mediated cell survival in ovarian cancer cells is associated with FoxO subcellular localization. Therefore, Mirk/Dyrk1B may be a novel target for treatment of ovarian cancer.

## Figures and Tables

**Table I:** Clinicopathological aspects and Mirk expression in patients.

Characteristic	No. of patients
Total	76
Age (years)^a^	47
Histology (positive for Mirk)^b^	
Cystadenocarcinomas	51 (38)
Serous	38 (31)
Mucinous	13 (7)
Cystadenomas	16 (6)^c^
Serous	9 (3)
Mucinous	7 (3)
Non-neoplastic cysts	9 (0)^d^

a Median age was 47 years (range = 32–64).

b Comparison of the incidence of cytoplasmic Mirk expression between subgroups.

c P<0.05 and

d P<0.0001 compared with cystadenocarcinomas.
